# Heart-lung transplantation using temperature- and airway pressure-controlled donor organ preservation system

**DOI:** 10.1016/j.xjtc.2026.102404

**Published:** 2026-05-08

**Authors:** Matthew M. Duda, Stefan Elde, Glenn J. Pelletier, Marc Leon, Katherine G. Verdi, Chawannuch Ruaengsri, Y. Joseph Woo, Yasuhiro Shudo

**Affiliations:** Department of Cardiothoracic Surgery, Stanford University School of Medicine, Stanford, Calif

**Figure:**
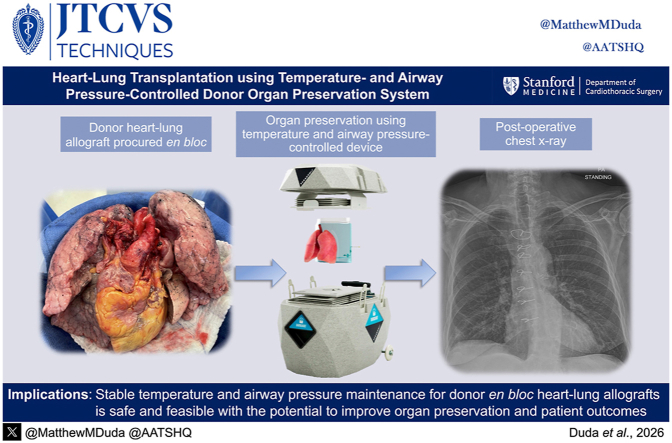

**Figure:**
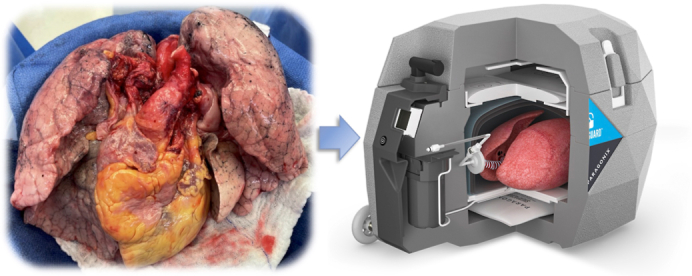
Temperature and pressure maintenance of heart-lung allografts is feasible and safe.

**Central Message**

The use of a temperature- and airway pressure-controlled donor organ preservation system for en bloc heart-lung transplantation is feasible and safe with the potential to improve patient outcomes.

Heart-lung transplantation represents a safe and effective treatment for end-stage combined heart and lung failure. Technologic advancements enabling stable temperature maintenance for donor heart and lung allografts may improve organ preservation and tolerance for cold ischemia, thus potentially increasing the donor pool.^1^ Lung allograft–preservation systems have been designed to maintain both temperature and airway pressure stability.^2^ However, no commercially available device has been specifically designed for use with a heart and lungs procured en bloc. Here, we present the safe application of a temperature- and airway pressure-controlled system, the Paragonix BAROguard, for en bloc heart-lung allograft preservation as a feasible approach to transplantation in patients with end-stage combined heart and lung failure.

## Case Presentation

The institutional review board of Stanford University approved the study protocol and publication of data. The patient provided written informed consent for the publication of the study data (institutional review board number: 80544, April 16, 2025). A 64-year-old female patient and former smoker with systemic sclerosis-related interstitial lung disease, chronic hypoxemic respiratory failure on 5 L/min home oxygen, and chronic systolic heart failure (left ventricular ejection fraction [LVEF] 25-30%) was admitted with acute-on-chronic heart failure. She was stabilized with inotropes and evaluated for heart-lung transplantation.

A suitable 53-year-old, female donor became available after cardiac arrest and declaration of brain death. The echocardiogram demonstrated LVEF 60 to 65%, normal right ventricular size and systolic function, and no hemodynamically significant valvular disease. Coronary angiography, imaging of the chest, and bronchoscopy were unremarkable. Arterial blood gas sampling showed arterial oxygen tension 548 mm Hg on 100% inspired oxygen fraction.

**Figure 1:**
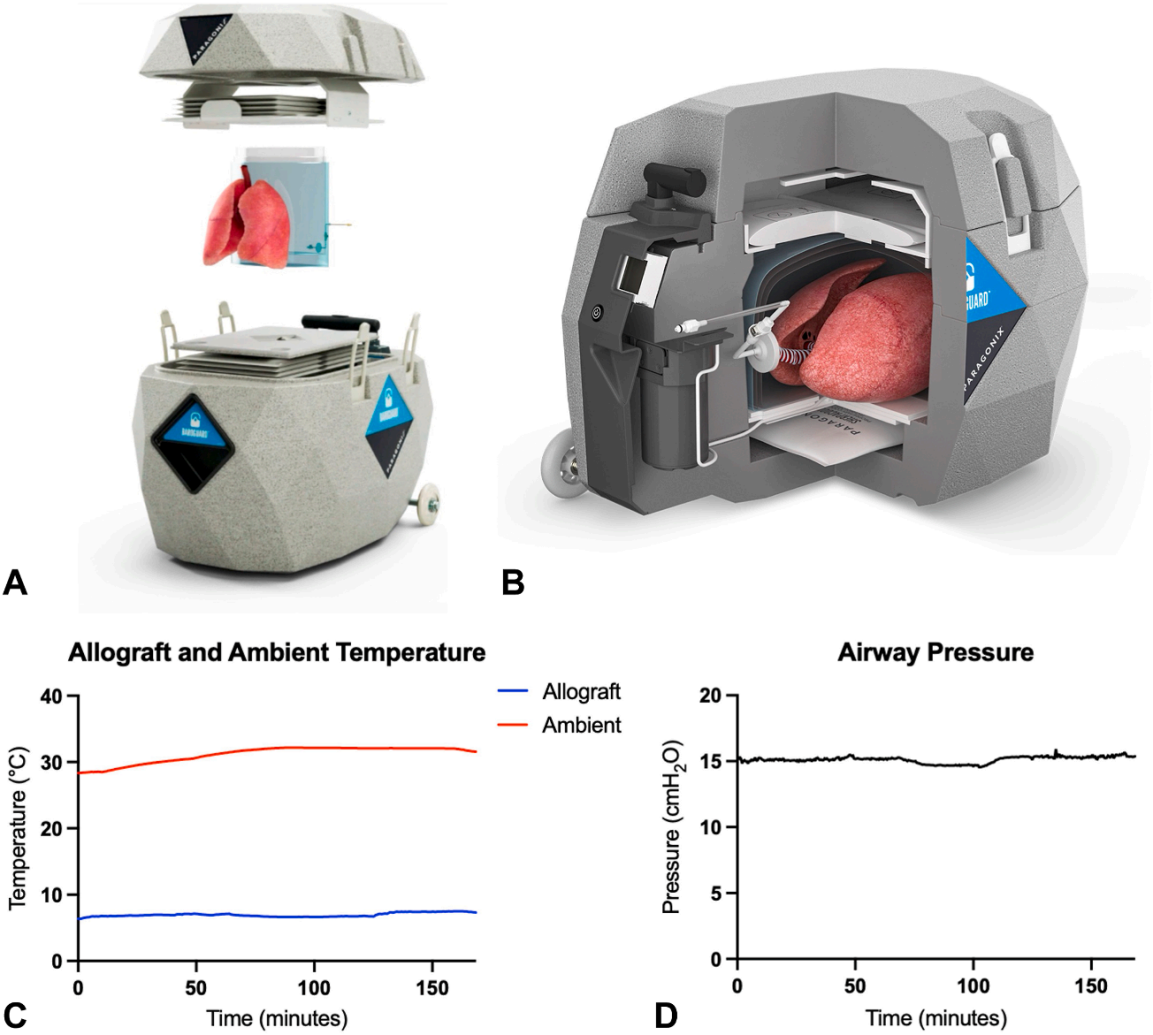
Organ-preservation system components separated (A) and combined (B). Temperature (C) and airway pressure (D) logs during transport.

Our team procured the heart-lung block using our standard technique.^3^ We then secured the tracheal connector to the trachea and placed the allograft into the inner bag within a set of 3 nested, sterile bags. We connected the attached tubing to the tracheal connector before releasing the tracheal clamp to maintain lung inflation. We then poured 2 L of ice-cold Physiolyte solution into the inner bag, deaired, and closed the bag securely. We poured 1 L of solution into the second bag and similarly secured it followed by the third bag without fluid. We lowered the bags containing the heart-lung block into the device and onto the temperature probe that measures the preservation fluid's temperature to approximate allograft temperature. Temperature is maintained by a proprietary phase-change material and insulation. We then connected the tracheal connector to the pressure regulator that actively maintains airway pressure between the recommended range of 12 to 15 cm H_2_O^2^ (Figure 1, *A* and *B*). The system recorded airway pressure and allograft and ambient temperatures during transport (Figure 1, *C* and *D*). The average allograft temperature was 6.97 °C, and the average ambient temperature was 31.2 °C. The average airway pressure was 15.1 cm H_2_O, which is similar to other published experiences with this device.^4^ The total travel distance was 370 miles, including a flight at >40,000-ft elevation. The total storage time was 173 minutes.

**Figure 2:**
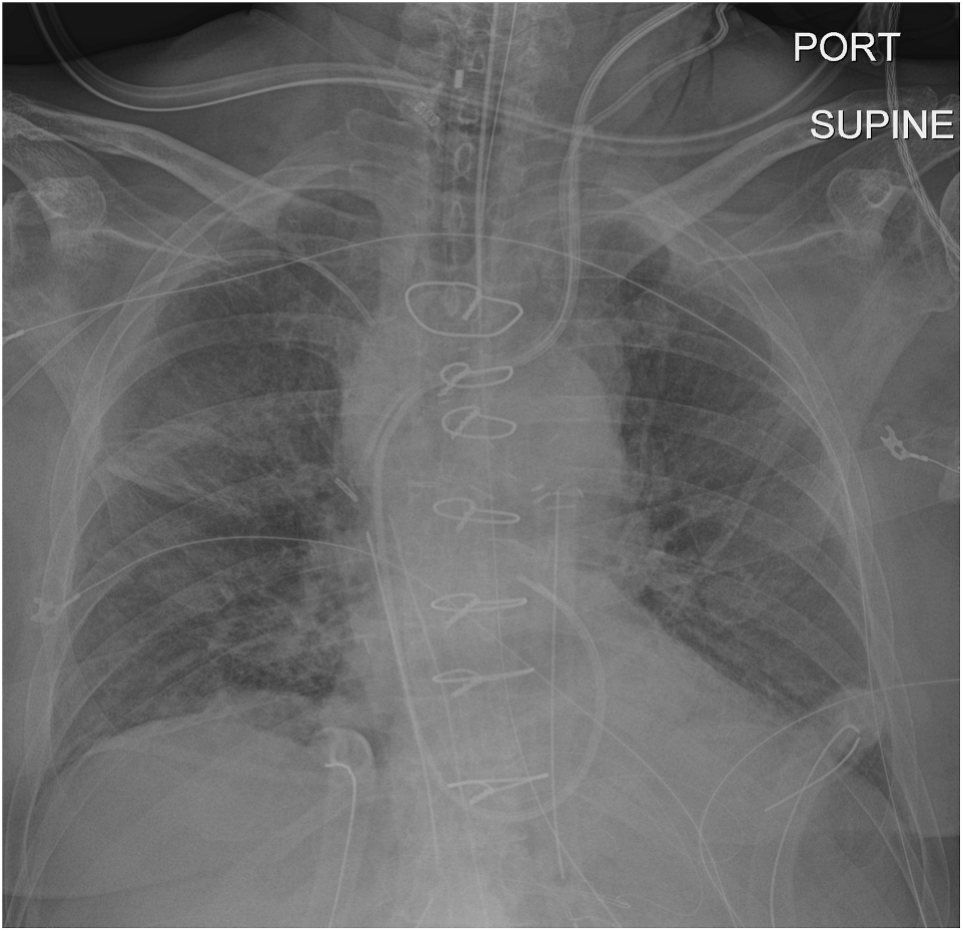
Radiograph of the patient’s chest on postoperative day 0 prior to extubation on postoperative day 1.

Recipient heart-lung explant and allograft implant were performed using standard techniques. Total ischemic time was 302 minutes; warm ischemic time was 52 minutes. The recipient cardiopulmonary bypass and aortic crossclamp times were 221 minutes and 166 minutes, respectively. The recipient recovered well without significant postoperative complication or need for mechanical circulatory support. She was extubated on postoperative day (POD) 1 with no evidence of pulmonary primary graft dysfunction (Figure 2). She initially required moderate-dose inotropes and demonstrated signs of mild cardiac primary graft dysfunction. Inotropes were weaned by POD 11, and she was transferred to the ward on POD 12 before discharging home on POD 22. At 1-year posttransplant, she continues to recover at home without signs of allograft dysfunction. A recent echocardiogram demonstrated normal biventricular function (LVEF 62.0%).

## Discussion

Heart-lung transplantation remains the only definitive therapy for end-stage combined heart and lung failure. However, organ demand continues to exceed supply for this life-saving treatment. To expand the pool of organs available for transplantation, improving preservation techniques is essential. Neto and colleagues^5^ reported the use of a temperature-controlled donor organ preservation system in en bloc heart-lung transplantation. Here, we describe the application of a temperature- and airway pressure-controlled system to heart-lung allograft preservation. Although the technique is safe and feasible, more investigation is necessary to determine how the device may impact patient outcomes in heart-lung transplantation.

## Conflict of Interest Statement

The authors reported no conflicts of interest.

The *Journal* policy requires editors and reviewers to disclose conflicts of interest and to decline handling or reviewing manuscripts for which they may have a conflict of interest. The editors and reviewers of this article have no conflicts of interest.
